# Orff-Based Music Training Enhances Children’s Manual Dexterity and Bimanual Coordination

**DOI:** 10.3389/fpsyg.2018.02616

**Published:** 2018-12-21

**Authors:** Marta Martins, Leonor Neves, Paula Rodrigues, Olga Vasconcelos, São Luís Castro

**Affiliations:** ^1^Center for Psychology at University of Porto, Faculty of Psychology and Education Sciences, University of Porto, Porto, Portugal; ^2^Centre of Research, Education, Innovation and Intervention in Sport, Faculty of Sport, University of Porto, Porto, Portugal; ^3^Research in Education and Community Intervention, Piaget Institute, Almada, Portugal

**Keywords:** music training, sports training, fine motor abilities, manual dexterity, bimanual coordination, children

## Abstract

How music training and expertise influence non-musical abilities is a widely researched topic. Most studies focus on the differences between adult professional musicians and non-musicians, or examine the effects of intensive instrumental training in childhood. However, the impact of music programs developed in regular school contexts for children from low-income communities is poorly explored. We conducted a longitudinal training study in such communities to examine if collective (Orff-based) music training enhances fine motor abilities, when compared to a homologous training program in sports (basketball), and to no specific training. The training programs in music and sports had the same duration, 24 weeks, and were homologous in structure. A pre-test, training, post-test and follow-up design was adopted. Children attending the 3rd grade (*n* = 74, 40 girls; mean age 8.31 years) were pseudorandomly divided into three groups, music, sports and control that were matched on demographic and intellectual characteristics. Fine motor abilities were assessed with the Purdue pegboard test (eye-hand coordination and motor speed, both subsumed under manual dexterity, and bimanual coordination) and with the Grooved pegboard (manipulative dexterity) test. All groups improved in manipulative dexterity that was not affected by type of training. On bimanual coordination and manual dexterity, however, a robust and stable advantage of music training emerged. At the end of training (post-test), children from the music group significantly outperformed children from the sports and control groups, an advantage that persisted at follow-up 4 months after training at the start of the following school year. Also, at follow-up none of the children from the music group were performing below the 20th percentile in the Purdue pegboard subtests and more than half were performing at the high end level (>80th percentile). Children from the sports group also improved significantly from pre- to post-test but their performance was not significantly different from that of the control group. These results show that an affordable, collective-based music practice impacts positively on fine-motor abilities, a finding that is relevant for a better understanding of the impact of music in childhood development, and that may have implications for education at the primary grade.

## Introduction

Music training is a powerful tool to study human behavior (Schellenberg, 2004). The effects of music training in school-aged children have deserved a lot of research attention, as childhood is a period of major developmental changes in cognitive, social and motor areas, and it is also when music training typically begins. When compared to control groups without music training, musically trained children have enhanced music-related skills, such as pitch discrimination (Ilari et al., 2016) and rhythm perception/production abilities (Matthews et al., 2016), and also advantages in non-musical domains, such as verbal abilities (Moreno et al., 2008, 2011; Degé and Schwarzer, 2011; Roden et al., 2012), executive functions (Degé et al., 2011; Moreno et al., 2011; Zuk et al., 2014), and even IQ (Schellenberg, 2004; Moreno et al., 2008; Degé et al., 2011). Less explored is how music training affects motor abilities. Of course, music practice involves motor actions whose characteristics are shaped, at least in part, by the instrument being played. However, there is presently no account that specifically addresses the impact of music training on the development of fine motor abilities.

Fine motor abilities allow us to make coordinated hand and finger movements to, for example, grasp and handle objects. They can be assessed with tasks that require one hand (unimanual; e.g., pencil grasp) or the coordinated activity of both hands (bimanual; e.g., knitting). Less effort is required performing with the preferred vs. the non-preferred hand, which is usually associated with slower and less accurate movements (Rodrigues et al., 2009; Serrien et al., 2014). Differences between hands are determined by hand preference and brain hemisphere specialization; using functional neuroimaging, Lavrysen et al. (2008, 2012) have shown that some left-right hand asymmetries depend on functional specialization of the left and right hemispheres that are independent from hand preference. In bimanual tasks both hands must work together even when the movements of each hand, or their role in the overall movement, differ, and so they are typically more demanding than unimanual tasks (Serrien et al., 2014). Interestingly, bimanual coordination can be strongly modulated by attentional focus. Deliberate attention to the non-preferred hand reduces left-right asymmetry (De Poel et al., 2008) and improves bimanual coordination (Pellegrini et al., 2004).

Fine motor abilities evolve over childhood. A developmental landmark occurs around 8 years, when a performance discontinuity related to changes in information processing capacities temporarily disturbs motor output (Serrien et al., 2014). Thus, the development of fine motor abilities interplays with the cognitive domain. An important instantiation of this is literacy acquisition. Writing requires eye-hand coordination and fine control of hand movements (Grissmer et al., 2010), and this motor component plays a non-negligible role in learning how to read and write. For example, Dinehart and Manfra (2013) have shown that object manipulation and motor writing ability were a strong predictor of 2nd-grade math and reading achievement of low-income children, even when controlling for demographic and cognitive characteristics.

It is a common belief that learning to play an instrument improves motor abilities. Indeed, continued practice of coordinated hand and finger movements together with attention to auditory feedback and online recalibration is bound to improve motor coordination and to fine-tune the integration of audio-visual information with motor control. However, studies addressing this issue are scarce and those that are relevant tend to compare adult musicians and non-musicians, leaving open the question of whether expertise-related differences reflect predispositions and/or result from training. An advantage of adult musicians when compared to non-musicians has been observed in finger-tapping tasks (Jäncke et al., 1997) and in unimanual and bimanual reaction times (Chang et al., 2014). Professional string players have a larger cortical representation of the fingers of the left-hand than non-musicians, early training associated with larger finger representation (Elbert et al., 1995); and professional musicians have larger anterior corpus callosum (indicative of better interhemispheric communication) than non-musicians, with more marked differences if training started before 7 years of age (Schlaug et al., 1995).

Extant studies with children have examined the effects of individual lessons of instrumental learning using longitudinal pre- and post-test designs. Costa-Giomi (2005) showed that some fine motor abilities of children who had 2 years of individual piano lessons improved significantly more than those of a control group of children with no training. The improvement was observed on the fine motor component of the Bruininks-Oseretsky Test of Motor Proficiency (BOT), specifically on the total score of this component and on the response speed subtest; no differences between groups were found in the visuo-motor coordination subtest nor in the upper-limb speed and dexterity subtest.

A positive effect of music training in fine motor abilities was also observed by Forgeard et al. (2008) in a study of 9-year-old children who had had three or more years of Suzuki or traditional instrumental instruction. Compared to a control group of children without music instrumental training, the musically trained 9-year-olds performed better in a four-finger sequence tapping task (completing three 4-finger sequences within 30 s). Note that both groups had 30- to 40-min weekly music classes in school, but these did not include instrumental training nor one-to-one tutoring. In a more recent study, Lampe et al. (2015) reported that 18 months of piano instruction, 30–45 min twice a week, significantly improved the uniformity of keystrokes of children and youths with hand motor disorders resulting from early brain damage. Converging evidence comes from a neuroimaging study by Schlaug et al. (2005) showing that musically trained children had significantly more gray matter in brain regions associated with skills learned during instrumental practice, namely independent fine motor control of both hands and auditory discrimination. Other behavioral studies suggest that early music instruction improves performance in domains associated with fine motor abilities, such as visuo–motor integration (Orsmond and Miller, 1999) and attentional capacity and reaction times (Patston et al., 2007).

Sports is another well-known activity that, as playing a musical instrument, is associated with improved motor abilities. The belief that those who practice sports or play a music instrument have enhanced motor abilities is widespread, and a few studies have approached this issue empirically. Correlational evidence has been collected in such diverse domains as movement timing, mental imagery, educational achievement, motivation and wellbeing. On timing skills, a comparison of adult athletes, musicians and controls revealed that although athletes outperformed musicians and controls in a circle-drawing task (emergent-based or continuous timing), in finger-tapping (event-based or discrete timing) both groups had similar precision but only musicians outperformed controls (Braun Janzen et al., 2014). On mental imagery, more specifically, left/right judgment, Dey et al. (2012) found no differences in accuracy and reaction times between children participating in music, sports, both or neither activity. With respect to impact on education, results from a survey conducted with teenagers from the German Socio-Economic Panel (Cabane et al., 2016) showed that, in comparison with doing sports, playing music correlated more with ambition and better academic performance, particularly for girls and for children coming from highly educated families; on the other hand, doing sports correlated more with better perceived health than playing music. Similarities between music and sports may extend to motivation and socio-affective dimensions (e.g., Martin, 2008). For example, children who spent more time in sports or music in elementary school were likely to have higher motivation to practice the same activity 4 years later (in adolescence), whereas children who had not participated in neither sports nor music were less likely to engage in these activities during adolescence (Simpkins et al., 2010). Importantly, socio-emotional wellbeing can benefit from practicing sports (Lubans et al., 2012), as well as from practicing music (Welch et al., 2014).

In the present study, our goal is to further investigate how fine motor abilities are influenced by music training, and sport will use the active control condition. We consider a different type of music training from the ones reviewed above: short-term (less than a year) Orff-based training in collective classes instead of relatively intensive individual lessons on how to play a musical instrument. In addition to a passive control group who will follow the regular school curriculum, our active control group will have sports training (basketball) of similar duration to the music training. Both training programs, music and sports, are conceived to be homologous and similarly challenging and attractive to the children; this allows to control for potential confounds due to motivational factors that would hinder an unambiguous interpretation of results (Moreno et al., 2008; Benz et al., 2016). Furthermore, the training programs will be conducted in a mostly low-income community as part of curricular and enrichment activities within the children’s regular school schedule. This provides an excellent model to study the potential impact of widespread music training, especially for under-privileged children (Kraus et al., 2014). The design of the study is longitudinal, with pre-test, training, post-test and follow-up phases. In light of the evidence reviewed above, we expect that music training leads to improved fine motor abilities, and specifically that Orff-based training impacts on bimanual coordination more than sports training or no training. Additionally, as both types of training, music and sports, involve visuo-motor components, such as eye-hand coordination and manual dexterity, we expect that children from those groups will have more gains in motor abilities than the control group.

## Materials and Methods

### Participants

Third graders were selected for this study because according to the Portuguese national curriculum collective sports and instrumental music are introduced as systematic school activities at this grade.

Eighty-six children were initially recruited to participate in the study. They were all Portuguese 3rd graders from five elementary public schools in the same greater Porto area in Northern Portugal. Children attending these schools come mostly from low-income communities: at the time of the study, more than 50% of them received social support from the Portuguese social security system and more than 70% of their parents had less than secondary education, a situation that was reflected in the SES of the children who participated in the study (see Table 1). The parents or legal guardians of the children completed a short questionnaire (see Procedure) from which we could gather that none of the children who were recruited had had prior experience in instrumental music practice nor in basketball practice. Twelve of them had to be excluded because of atypically low full-scale IQ (below 70; World Health Organization, 1992; *n* = 7), neurological problems (*n* = 1), school transfer (*n* = 2) and incomplete data records (*n* = 2). The final sample consisted of 74 children (34 boys and 40 girls, *M* age = 8.31 years, range = 7.75–9.50, *SD* = 0.35). Four were left handed, according to the Edinburgh Handedness Inventory (Oldfield, 1971), and 42 were involved in some kind of extra-curricular activity outside of the school, mostly sports other than basketball (either football or swimming). The children were assigned to the music, sports (active control) or no training (standard control) groups such that these were matched on age, full-scale IQ, verbal IQ and performance IQ (all *F*s < 1). The groups also did not differ on sex [χ^2^_(2)_ = 0.26, *p* = 0.88], socioeconomic status [χ^2^_(2)_ = 2.55, *p* = 0.28] and handedness (*F* < 1). One of the left-handed girls was in the music group, the other in the control group, and one of the left-handed boys was in the sports group, the other in the control group. The groups were also similar regarding participation in extra-curricular activities not related to the study: 10 in the music group, 16 in the sports group and 16 in the control group [χ^2^_(2)_ = 4.36, *p* = 0.11].

**Table 1:** Demographic, cognitive and motor characteristics of children in the music, sports, and control groups prior to training.

Characteristics	Music group *n* = 25	Sports group *n* = 25	Control group *n* = 24
Sex	13 F/12 M	13 F/12 M	15 F/9 M
SES^a^	14 LM–/11 M+	11 LM/14 M+	8 LM/16 M+
Age in years	8.35 ± 0.31 (7.83–9.00)	8.29 ± 0.42 (7.75–9.50)	8.29 ± 0.31 (7.83–8.75)
Handedness^b^	84.80 ± 29.53 (−35–100)	83.80 ± 30.25 (−40–100)	71.25 ± 55.37 (−100–100)
Full-scale IQ^c^	95.44 ± 12.32 (74–113)	93.60 ± 13.40 (74–120)	96.29 ± 14.73 (74–125)
Verbal IQ	97.00 ± 14.89 (69–124)	94.72 ± 11.96 (72–116)	95.58 ± 13.43 (70–120)
Performance IQ	97.16 ± 11.59 (75–118)	96.08 ± 13.89 (70–119)	99.08 ± 15.28 (74–132)
**Purdue pegboard test**^d^			
Preferred hand	24.80 ± 3.37 (18–30)	23.60 ± 3.92 (16–30)	25.17 ± 3.38 (20–32)
Non-preferred hand	22.64 ± 2.50 (16–26)	21.92 ± 2.97 (16–28)	23.08 ± 4.41 (14–32)
Both hands	18.08 ± 3.19 (12–24)	16.88 ± 3.32 (10–24)	18.67 ± 3.85 (8–28)
**Grooved pegboard test**^d^			
Preferred hand	18.16 ± 3.60 (9–25)	16.62 ± 3.87 (10–26)	18.37 ± 3.72 (10–26)
Non-preferred hand	16.78 ± 3.79 (10–25)	14.57 ± 3.48 (8–22)	16.36 ± 4.11 (7–24)

*Range in parentheses. ^a^SES as used in the Portuguese public school system: low or middle low (LM−) if children get free or price-reduced school meals, and middle or higher (M+) if no price reduction in school meals; ^b^Edinburgh Handedness Inventory; ^c^Wechsler Intelligence Scale for Children – WISC-III, Portuguese version; ^d^pegs/min.*

The study was approved by the ethics committee of the Faculty of Psychology and Education Sciences at University of Porto and the school boards. Written informed consent was obtained from parents or legal guardians of the children, who gave their verbal assent before data collection started.

### Design and Materials

This is a longitudinal training study that consisted of a pre-test, training for 24 weeks, a post-test and a follow-up 4 months after the end of training. In the pre-test phase, children completed an assessment protocol including handedness, general intellectual ability and fine motor abilities. In the post-test and follow-up phases, their motor abilities were again tested.

Handedness was assessed with the Edinburgh Handedness Inventory (Oldfield, 1971), and general intellectual ability with the Portuguese version of the Wechsler Intelligence Scale for Children - 3rd Edition (WISC-III; Wechsler, 2003). All of the WISC-III subtests required to compute verbal IQ, performance IQ and full-scale IQ were used, viz. the Information, Similarities, Arithmetic, Vocabulary, and Comprehension subtests (verbal IQ) and the Picture Completion, Coding, Picture Arrangement, Block Design, and Object Assembly subtests (performance IQ). Fine motor abilities were assessed with two well established tests, the Purdue Pegboard test (Tiffin, 1968) and the Grooved Pegboard test (Trites, 1989).

The Purdue Pegboard test provides a measure of manual dexterity and bimanual coordination that relies on eye-hand coordination and motor speed. It consists of a board with two vertically aligned series of 25 holes where as many pegs as possible have to be inserted within a time limit of 30 s. This task is performed with the preferred hand and with the non-preferred hand (unimanual subtests) and with both hands simultaneously (bimanual subtest). In the unimanual subtests, the score is the number of correctly inserted pegs, and in the bimanual subtest it is the number of pairs of pegs. In order to make results directly comparable across the two pegboard tasks (see below), we considered the ratio of pegs over time and used the number of pegs (or peg pairs) per minute as the measure of motor performance. The Grooved Pegboard test also requires participants to insert pegs on a board, but the holes are shaped in different orientations (key holes) and the pegs must be rotated accordingly in order to enter the key hole. This test provides a measure of manipulative dexterity that taxes visuo-spatial processing in addition to eye-hand coordination and motor speed. The board consists of a 5 × 5 matrix of key holes with different orientations, and the task is to be performed unimanually with the preferred hand and with the non-preferred one. The instruction is that the pegs should be inserted as quickly as possible. Based on age-related norms (Trites, 1989), we required children to fill the two first rows of the pegboard (2 × 5 matrix; total of 10 pegs) with each hand, as quickly as possible. As the dependent measure, we took the number of pegs inserted per minute.

### Training

The music and basketball training programs were prepared specifically for this study with the collaboration of two professional age-appropriate teachers, one specialized in music and the other in sports/basketball. Both programs consisted of structured groups of learning activities adapted to elementary school children with no prior systematic music or basketball instruction, and were organized to fit into the schedule of regular and extra-curricular (enrichment) school activities as two 90-min collective sessions per week. They were conceived to be analogous regarding difficulty, expected progression along time and motivational aspects; for example, both programs included public presentations (musical performance/basketball game) for school and local communities. Thanks to an agreement protocol with local school authorities, the programs were provided to the children in the context of their school attendance and no extra fee was required.

The music training program used an Orff-based approach to initiate children into music knowledge and skill. The program was structured into four main areas: music awareness, elementary music concepts, rhythm and pitch skills, and instrumental and vocal performance (see Supplementary Table S1). Auditory and conceptual work was combined with collective Orff-type music practice such that music concepts were taught and practiced through instrumental playing, singing, or movement. Activities consisted mainly of collective instrumental practice using descant recorder, drums, xylophones and metallophones; singing, body percussion and unpitched percussion instruments were also included, though less frequently. In order to create challenging sound environments, all activities were planned to include at least three different sound sources. Children tried out various Orff instruments for a given music piece, and more complex melodic and rhythmic patterns were introduced according to their progress.

The sports training program consisted of basketball practice, including technical knowledge and skill. Activities were also organized into four areas: physical fitness, game relevant motor coordination (upper and lower limbs, eye-hand coordination), team sports concepts and schemes, and tactical planning (see Supplementary Table S2). Physical fitness activities and coordination exercises progressed from general to basketball-oriented. Training emphasized the development of coordination skills and visuo-spatial performance as an individual player, but also at the collective level as member of a team.

Each of the training programs was conducted by the same teacher throughout its entire duration. The music teacher was graduated in Music Education and a regular chamber orchestra practitioner. The sports teacher had a degree in Physical Education and Sports and was a professional basketball team coach. Both of them had more than 10 years of professional experience with elementary school children.

### Procedure

Prior to the start of data collection, the children’s parents completed a questionnaire on demographic characteristics, on their children’s previous experience regarding music and sports, namely basketball, training, and also on other current extra-curricular activities they might have been engaged with. Information regarding support received from the national social security system was also gathered, that is, whether children had the right to free or price-reduced school meals, or if no such reduction was applicable. This was used as a proxy for socioeconomic status that was classified as low or middle-low in the former case, and middle or higher in the second case.

At pre-test, post-test and follow-up, children were individually assessed in a quiet room of their school. The WISC-III battery was administered in one session by an experienced child psychologist. The handedness and motor tests were completed in different sessions and were administered by a trained research assistant. In each of the study phases, half of the children of each group started with the Purdue pegboard test and the other half with the Grooved pegboard test. The order of the hand (preferred vs. non-preferred) was counterbalanced in each group and test. Bimanual performance of the Purdue pegboard test was always assessed after both Purdue unimanual subtests. To ensure that the instructions had been properly understood, children were given a training trial of each test and subtest. Also, to exclude potential artifacts due to stress, time only started counting when the child picked up the first peg (Hermsdörfer et al., 1999). During unimanual tasks, the unused hand was placed over the table to the side of the pegboard.

Both training groups started their music and basketball sessions after pre-test assessment at the start of the school year (October). Training took place twice a week in 90-min sessions and lasted for almost a school year, from October until May with interruptions for school holidays – so in practice, there were 24 weeks of training. Children in the music group gave a public performance at the end of the school year, and children in the sports group participated in a basketball tournament. Children in the standard control group were engaged in different types of extra-curricular school activities not including systematic music or basketball training. Both types of training were planned to be available in the following year so that interested children from the standard control group could participate in them. Finally, children from the three groups completed a follow-up assessment 4 months later, at the beginning of the next school year.

## Results

**FIGURE 1:**
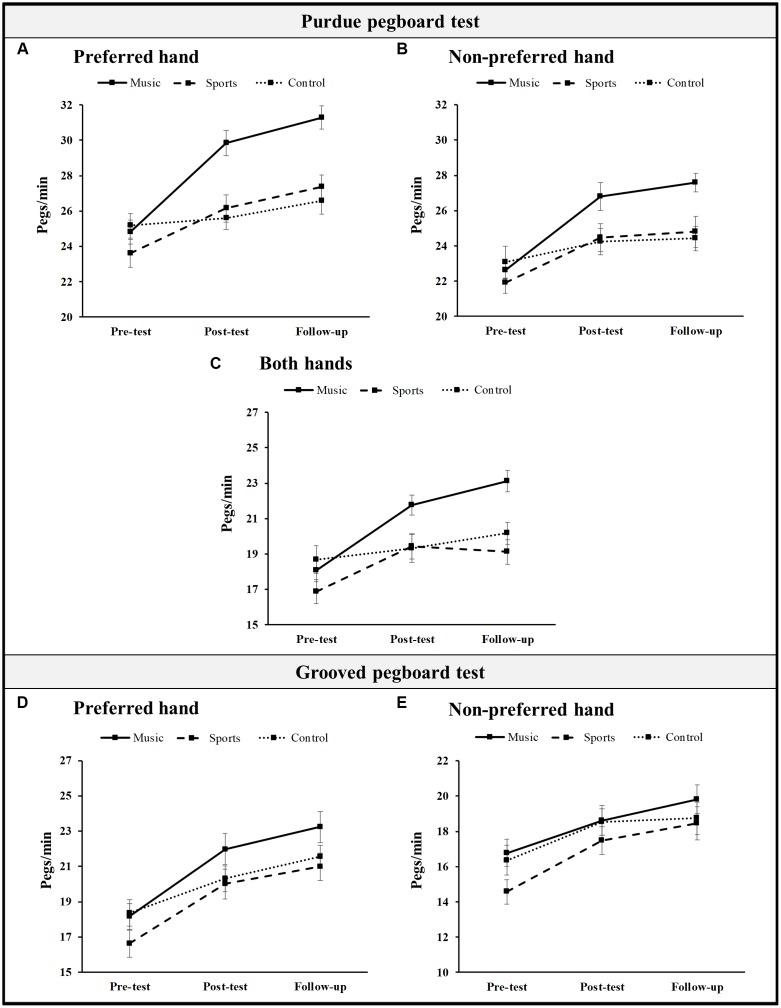
Mean scores (pegs per minute) in the Purdue Pegboard test with the preferred hand **(A)**, non-preferred hand **(B)**, or both hands **(C)**, and in the Grooved pegboard test with the preferred hand **(D)**, and non-preferred hand **(E)**, for the music, sports, and control groups at pre-test, post-test, and follow-up. Error bars indicate SEM.

The results obtained for each group in the motor performance tests before and after training including follow-up are shown in Figure 1. In a set of preliminary analyses, we checked if there were no group differences in motor skills prior to training, and confirmed that there were none: for the Purdue pegboard test with the preferred hand, *F*_(2,71)_ = 1.30, *p* = 0.28, with the non-preferred hand, *F* < 1, and with both hands, *F*_(2,71)_ = 1.71, *p* = 0.19; for the Grooved pegboard test with the preferred hand, *F*_(2,71)_ = 1.62, *p* = 0.21, and the non-preferred hand, *F*_(2,71)_ = 2.38, *p* = 0.10. We then analyzed the effects of training by calculating repeated measures ANOVAs with Group (music, sports and control) as between-subjects factor and Time (pre-test, post-test and follow-up) as within-subjects factor. Differences between groups at each time point and progression across time points were tested using *post hoc* pairwise-comparisons with the Bonferroni correction. For each pairwise comparison we report the mean difference *M*, standard error *SE*, *p*-value and Cohen’s *d* effect size.

Performance on the Purdue pegboard test with the preferred hand improved across time (main effect of Time: *F*_(2,142)_ = 44.45, *p* < 0.001, η_*p*_^2^ = 0.39), with a significant increase from pre- to post-test (*M* = 2.67 pegs/min, *SE* = 0.44, *p* < 0.001, *d* = 0.75) and from post-test to follow-up (*M* = 1.21 pegs/min, *SE* = 0.39, *p* < 0.01, *d* = 0.35). The main effect of Group was also significant, *F*_(2,71)_ = 8.69, *p* < 0.001, η_*p*_^2^ = 0.20: the music group outperformed both the sports (*M* = 2.93 pegs/min, *SE* = 0.80, *p* = 0.001, *d* = 1.03) and the control (*M* = 2.86 pegs/min, *SE* = 0.81, *p* < 0.01, *d* = 1.01) groups. More important to the present study, the interaction Time x Group was also significant, *F*_(4,142)_ = 7.35, *p* < 0.001, η_*p*_^2^ = 0.17. As is clear from Figure 1A, the greatest increase from pre-test to post-test occurred in the music group, *M* = 5.04 pegs/min, *SE* = 0.76, *p* < 0.001, *d* = 1.45. The sports group also improved significantly from pre- to post-test, but not so markedly: *M* = 2.56 pegs/min, *SE* = 0.76, *p* < 0.01, *d* = 0.66. In the control group, the increase was not significant (*M* = 0.42 pegs/min, *SE* = 0.77, *p* = 1.00, *d* = 0.13). Differences from post-test to follow-up were not significant in either group (*p_s_* > 0.05). Looking at differences between groups, the same pattern of superiority of music training emerges: the music group significantly outperformed the sports group at post-test, *M* = 3.68 pegs/min, *SE* = 0.99, *p* = 0.001, *d* = 0.99, and follow-up, *M* = 3.92 pegs/min, *SE* = 0.99, *p* = 0.001, *d* = 1.17, and the control group at post-test, *M* = 4.26 pegs/min, *SE* = 1.00, *p* < 0.001, *d* = 1.29, and follow-up, *M* = 4.70 pegs/min, *SE* = 1.00, *p* < 0.001, *d* = 1.33.

With the non-preferred hand, performance on the Purdue pegboard test showed a significant main effect of Time, *F*_(2,142)_ = 34.85, *p* < 0.001, η_*p*_^2^ = 0.33, and an interaction of Group with Time, *F*_(4,142)_ = 3.98, *p* = 0.004, η_*p*_^2^ = 0.10; the main effect of Group was barely significant [*F*_(2,71)_ = 3.14, *p* = 0.05, η_*p*_^2^ = 0.08]. More specifically, performance improved from pre- to post-test, *M* = 2.63 pegs/min, *SE* = 0.41, *p* < 0.001, *d* = 0.73, but not from post-test to follow-up, *M* = 0.43 pegs/min, *SE* = 0.38, *p* = 0.77, *d* = 0.11; both the music group and the sports group improved significantly from pre-test to post-test, but the improvement was greater in the music group, *M* = 4.16 pegs/min, *SE* = 0.71, *p* < 0.001, *d* = 1.27, than in the sports group, *M* = 2.56 pegs/min, *SE* = 0.71, *p* < 0.01, *d* = 0.73. There were no significant differences in the control group from pre- to post-test (*M* = 1.17 pegs/min, *SE* = 0.72, *p* = 0.33, *d* = 0.29). Neither group had significant improvements from post-test to follow-up (*p_s_* > 0.05). Turning now to differences between groups, the only significant ones were at follow-up, where the music group outperformed the sports group, *M* = 2.80 pegs/min, *SE* = 1.01, *p* = 0.02, *d* = 0.77, and the control group, *M* = 3.18 pegs/min, *SE* = 1.02, *p* < 0.01, *d* = 1.04.

Bimanual performance on the Purdue pegboard test showed significant main effects of Time, *F*_(2,142)_ = 30.60, *p* < 0.001, $\eta_{p}^{2}$ = 0.30, Group, *F*_(2,71)_ = 5.50, *p* = 0.006, η_*p*_^2^ = 0.13, and the double interaction, *F*_(4,142)_ = 4.68, *p* = 0.001, $\eta_{p}^{2}$ = 0.12. As in the unimanual subtests, improvements from pre- to post-test occurred in the music group, *M* = 3.68 pegs/min, *SE* = 0.69, *p* < 0.001, *d* = 1.24, and in the sports group, *M* = 2.56 pegs/min, *SE* 0.69, *p* < 0.01, *d* = 0.75, but not in the control group (*M* = 0.67 pegs/min, *SE* = 0.71, *p* = 1.00, *d* = 0.17); between post-test to follow-up, there were no improvements (*p_s_* > 0.05). Between-group comparisons again showed a superiority of the music group, that outperformed the sports group at follow-up, *M* = 4.00 pegs/min, *SE* = 0.89, *p* < 0.001, *d* = 1.24, and the control group at post-test, *M* = 2.43 pegs/min, *SE* = 0.97, *p* = 0.04, *d* = 0.73, and follow-up, *M* = 2.95 pegs/min, *SE* = 0.90, *p* = 0.005, *d* = 0.99. A barely significant advantage of the music group when compared to the sports group was also observed at post-test, *M* = 2.32 pegs/min, *SE* = 0.96, *p* = 0.06, *d* = 0.73.

Performance on the Grooved pegboard test had a different pattern of results. In the subtest with the preferred hand, only Time had a significant effect, *F*_(2,142)_ = 80.92, *p* < 0.001, $\eta_{p}^{2}$ = 0.53; neither Group [*F*_(2,71)_ = 1.88, *p* = 0.16, $\eta_{p}^{2}$ = 0.05] nor the interaction Time x Group [*F*_(4,142)_ = 1.81, *p* = 0.13, $\eta_{p}^{2}$ = 0.05] reached significance. There was an increase from pre- to post-test, *M* = 3.04 pegs/min, *SE* = 0.34, *p* < 0.001, *d* = 0.78, and from post-test to follow-up, *M* = 1.17 pegs/min, *SE* = 0.34, *p* < 0.01, *d* = 0.29. With the non-preferred hand, again only the effect of Time was significant, *F*_(2,142)_ = 41.82, *p* < 0.001, $\eta_{p}^{2}$ = 0.37; performance increased from pre- to post-test, *M* = 2.29 pegs/min, *SE* = 0.32, *p* < 0.001, *d* = 0.59, and, barely significantly, from post-test to follow-up, *M* = 0.80 pegs/min, *SE* = 0.35, *p* = 0.07, *d* = 0.19. Neither Group [*F*_(4,142)_ = 1.18; *p* = 0.31; $\eta_{p}^{2}$ = 0.03] nor the interaction reached significance [*F*_(4,142)_ = 1.02, *p* = 0.40, $\eta_{p}^{2}$ = 0.03].

**FIGURE 2:**
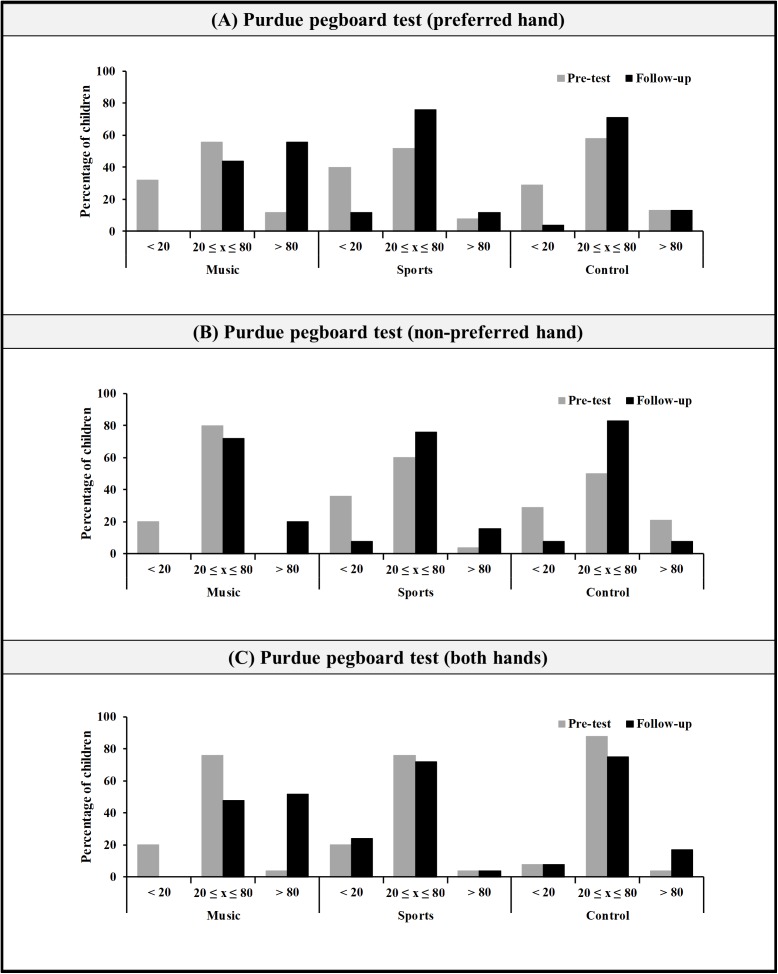
Distribution of children from the three groups according to performance levels: low (<20th percentile), middle (20th–80th percentile), and high (>80th percentile), in the Purdue pegboard test with the preferred hand **(A)**, non-preferred hand **(B)**, and both hands **(C)**.

In order to take a closer look at how training might have affected performance, we examined the distribution of individual results across performance levels. This analysis only considered the subtests showing the interaction of Time with Group (the Purdue subtests). Furthermore, we compared directly pre-test with follow-up as there was no evidence of performance changes from post-test to follow-up. Performance levels were defined in accordance with the normative values provided by Gardner and Broman (1979): below the 20th percentile (low), between the 20th and the 80th percentiles (middle), and above the 80th percentile (high). The distribution of children from the music, sports and control groups as a function of performance level at pre-test and follow-up is illustrated in Figure 2. At pre-test, there were no significant differences between groups on the proportion of children in each performance level in the Purdue preferred hand [χ^2^_(2)_ = 0.83, *p* = 0.93, *V* = 0.11] and bimanual [χ^2^_(2)_ = 1.63, *p* = 0.80, *V* = 0.15] subtests; with the non-preferred hand, the control group had a greater proportion of high-performing children than the music group, χ^2^_(2)_ = 10.28, *p* = 0.04, *V* = 0.37. At follow-up, a significantly higher proportion of children from music group reached a high performance level when compared to sports and control groups, both in the preferred hand, χ^2^_(2)_ = 17.90, *p* = 0.001, *V* = 0.49, and bimanual subtests, χ^2^_(2)_ = 21.23, *p* < 0.001, *V* = 0.54. Although no significant differences were observed in the proportion of children from each group in the Purdue non-preferred hand subtest [χ^2^_(2)_ = 5.00, *p* = 0.29, *V* = 0.26], almost thirty percent (28%) of the children from the music group reached the high-performing level, compared to 16% from the sports group and 8% from the control group. None of the children from the music group scored at a low performance level in any of the Purdue subtests.

## Discussion

The present study investigated how children’s fine motor abilities are influenced by music training. Unlike previous studies that relied on instrumental music instruction for at least 2 years, we focused on collective Orff-based music practice for a relatively short duration (24 weeks), and included an active control group undertaking basketball training in addition to a standard control group. Children were 8-year-olds attending the 3rd grade of public schools from mostly low-income communities. We measured eye-hand coordination, motor speed and bimanual coordination with the Purdue pegboard test, and manipulative dexterity with the Grooved pegboard test, before training, immediately after training finished at the end of the school year, and at the start of the following school year (follow-up). Overall, we found that children from the three groups improved in their motor abilities from pre-test to follow-up, but progress was not of the same magnitude for all of them: it depended on type of training and on the type of test. Critical to the goals of this study, there was an advantage of music training over sports training or no-training in enhancing bimanual coordination and manual dexterity that persisted at follow-up 4 months later. Sports training was also associated with significant progress across test points. These main findings (music training advantage; the case of sports training; overall progress) will be discussed below.

The advantage of music training showed up not only in bimanual coordination, as we had expected on the basis of the characteristics of Orff-based music practice, but also in manual dexterity. Musically trained children outperformed children from the sports and control groups at post-test and follow-up in the Purdue pegboard test with both hands (bimanual coordination) and with the dominant hand (manual dexterity). In both cases, *d* effect sizes ranged from 0.73 to 1.29 at post-test and were slightly greater at follow-up, from 0.99 to 1.33. Furthermore, at follow-up most children from the music group were performing at the upper level (80th percentile and up), and none in the lowest 20th percentile. With the non-dominant hand, progress from pre-test to post-test was also more marked in the music group (*d* = 1.27) than in the sports (*d* = 0.73) and the control (*d* = 0.29, *ns*) groups, but interestingly it was only at follow-up that significant between-groups differences emerged, with an advantage of the music over the sports group with a *d* of 0.77 and over the control group with a *d* of 1.04. These findings clearly indicate that the effect of music training in enhancing manual dexterity and bimanual coordination were not a short-lived consequence of having played Orff instruments in the previous month or so. It is particularly revealing that the superiority of music training was maintained after 4 months, namely because this included summer holidays and thus an interval of almost 2 months from the typical school-like activities involving fine motor activities such as writing. We interpret this finding as indicating the stability of the improvement in fine motor abilities elicited by music training.

The findings described above come from the Purdue pegboard test and do not extend to the Grooved pegboard test that has a strong component of manipulative dexterity. Indeed, both tests involve different movement subcomponents: picking up the peg, moving the peg to the hole, placing the peg in the hole, and moving the hand to pick up the next peg (Roy et al., 2003), but critical to the Grooved pegboard test is visuo-spatial processing and manipulative ability that as such are not required, or are secondary, in the Purdue pegboard test. In the Purdue pegboard test, the third sub-component accounts for the largest amount of motor task time, and is probably the one that most aptly captures a developmental parameter (e.g., Annett et al., 1979). It is also likely that it is the one that accounts for our results as it is most directly related to Orff-based training. Apart from bimanual coordination, Orff-based music practice involves eye-hand coordination and the ability to integrate speed and precision with controlled discrete movements, that is, event-based timing mechanisms (Baer et al., 2013; Braun Janzen et al., 2014). For instance, drumming, playing the xylophone or playing drones^1^, all three afford training opportunities to hit a target, maintain timing and movement accuracy, and coordinate both hands or upper limbs (in drone execution, both arms move synergistically for a relatively extended period of time). The placement of the peg may be considered homologous to ‘hit the target’ in Orff practices where, as in the Purdue pegboard test, it is not preceded by a manipulative movement. So it is possible that the effects that we have found on the Purdue pegboard test are an expression of near transfer from Orff-based music training to bimanual coordination, eye-hand coordination, and motor speed.

Another possibility is that the music advantage is not driven solely by movement-related processes, and that the benefits to fine motor skills arise via attentional mechanisms that are an important facet of music training (Duke et al., 2011; Strait et al., 2015). Music practice is a multisensory motor experience in which the player integrates sequential movements into a rhythmic and expressive context. The refinement of the movement may be achieved not only through repetition, or practice, proper, but also as a result of (or concomitantly with) the auditory feedback that allows for precise timing of motor control and gradually shapes performance (Schneider et al., 2010; Bevilacqua et al., 2016). The notion that music training is associated with enhanced control of attention has received empirical support from behavioral and neuroimaging studies (e.g., Gaser and Schlaug, 2003). For example, in comparison with non-musicians, orchestra musicians have better performance on selective, divided, and sustained attention tasks (Rodrigues et al., 2014), and a more balanced visual attentional capacity (Patston et al., 2007). However, as we did not manipulate attention our findings do not allow us to disentangle between potential motor-based or attention-based processes as the primary mechanism leading to the advantage of music training for fine motor abilities. This is a question to be addressed in future studies.

Irrespective of the mechanisms subtending the observed effects, our finding of an improvement in children’s fine motor abilities following music practice agrees with previous ones from Costa-Giomi (2005) and Forgeard et al. (2008) studies. Interestingly, fine motor abilities were measured differently in each of these studies and the improvement that was observed had some degree of specificity. In Costa-Giomi’s study, it was response speed subtests, and not visuo-motor coordination or dexterity subtests, that carried the effect of the piano lessons; and in Forgeard et al.’s study, children had been learning how to play keyboard and/or string instruments and what was measured was finger independence and coordination, a certainly trained component during this sort of instrumental practice. In our study, the improvement that was specific to the music group included bimanual coordination, and eye-hand coordination and response speed, but not manipulative dexterity. So music training appears to induce improvement of fine motor abilities whose characteristics are either basic and general (motor speed) or linked to the music practiced (finger independence, bimanual coordination). Another important conclusion that may be drawn from the comparison of these studies and ours is that music training does not need to be of long duration nor in the form of individual classes of instrumental instruction to impact on fine motor abilities: collective Orff-based music practice for 24 weeks was sufficient to bring about positive effects that were stable for at least 4 months after training.

The second set of results worth mentioning concerns sports training, which in this study consisted of basketball. This sport involves gross motor activities like running and jumping that hardly bear any similarity with music training, but it also includes eye-hand coordination, bimanual coordination and manual dexterity (e.g., Park et al., 2011; Tsang et al., 2014) that resemble some of the aspects of music training. Additionally, both sports and music are bound to have a positive influence in a variety of domains (e.g., Martin, 2008; Cabane et al., 2016) including well-being (Lubans et al., 2012; Welch et al., 2014). So it is not surprising that, as we had expected, the sports group improved significantly from pre-test to post-test in the same abilities as the music group. However, the effect sizes were smaller (>1 in the music group, around 0.7 in the sports group). In between-group comparisons the advantages of the sports group over the control group were not significant, whereas the ones from the music group were. These results show that basketball training was not as effective as music training in improving children’s fine motor abilities. It is possible that in order to elicit substantial, or more marked, training effects sports and music require different amounts of practice, and/or have different time courses. If that is the case, longer or more intensive training in basketball might be necessary to achieve similar effects as music training. That music and sports/movement training may have homologous effects, with an advantage to music in some cases, is indeed what has been recently found in studies of science-based music rehabilitation of motor impairment (Moumdjian et al., 2017). For example, Schneider et al. (2010) have shown that music training was more effective than a functional motor program for the recovery of motor impairments in stroke patients. Findings from timing entrainment also suggest that music might have a more a powerful effect than sports, at least in the sense that music expertise boosts both event- and emergent-timing whereas the benefits from sports expertise appear to be confined to emergent timing (Braun Janzen et al., 2014). This might be related to different weightings that movement and flow, on the one hand, and finer-grained vs. coarser-grained events, on the other hand, might have in doing sports in comparison with playing music: maybe timing and control of fast paced and fine-grained events (such as key presses in playing piano) are more critical to music than to sports. However, level of expertise and specific characteristics of the type of sport and music under comparison would also have to be examined in order to move from speculation to established finding. Our study focused on a relatively narrow set of fine-motor abilities, and thus it does not allow us to make that step. Indeed, a limitation of this study is to not have included the assessment of gross motor abilities that might be closer to basketball training than the fine motor abilities examined.

The final and third set of results is progress from pre-test to post-test and follow-up. In all subtests of the Purdue and Grooved pegboard tests we found a significant effect of time that suggests a pattern of general improvement in fine motor abilities. This was particularly marked in the tests of manipulative dexterity (Grooved pegboard test), where no interaction with group and no specific effects of music or sports training were found. Although direct comparisons of effects sizes from different tests should be interpreted with caution, it is interesting that the improvement from pre-test to post-test in manipulative dexterity was greater than that of the control group in manual dexterity (Purdue pegboard test). This apparently stronger progress in manipulative dexterity across time is consistent with Serrien et al.’s (2014) suggestion of heterogeneity in the development of motor abilities, where certain of them have a plateau at around 8 years of age, whereas others continue to improve. This would be the case of our findings in the Grooved pegboard test, where the progress from pre- to post-test and follow-up was not linked to specific training and is likely due to the development in manipulative ability that occurs at this age. However, the design of our study does not allow to firmly conclude on this issue. It should also be addressed in future studies.

Summing up, the present study has shown that collective Orff-based music training improves children’s fine motor abilities, namely bimanual coordination and manual dexterity. To the best of our knowledge, this is the first time that this type of music training effect was given a stringent test by adopting a longitudinal approach with an active control group of basketball training as well as a standard passive control group. Importantly, training was embedded in regular school activities, and children came from mostly low-income communities. As such, the findings from the present study open an exciting prospect for educational contexts. Music-related benefits can be achieved at a good cost/benefit ratio, and may be especially relevant for children from low-income communities who often do not have the opportunity to be part of these activities outside of school. School-based programs may thus be an important means for foster the academic trajectory and the quality of life of all children (e.g., Eerola and Eerola, 2014; Kraus et al., 2014; Welch et al., 2014). As stressed by Valencia (2010), structural measures are especially valuable to reduce social inequality without falling prey to a deficit model of development where low-income might be inadvertently equated with at-risk. Training programs such as the one presented in this study may thus contribute to the ongoing discussion about educational practices suitable for all.

## Author Contributions

SLC and MM designed the study. MM and LN collected the data. PR, OV, and SLC supervised data-analysis and interpreted the data. MM analyzed and interpreted the data. SLC, MM, and LN wrote the manuscript. All authors read and approved the final version of the manuscript.

## Conflict of Interest Statement

The authors declare that the research was conducted in the absence of any commercial or financial relationships that could be construed as a potential conflict of interest.

## Footnotes

1 A drone is a harmonic or monophonic effect or accompaniment where a note, a set of notes or a chord is continuously sounded throughout most or all of a piece.
