# Supplementary material for: Orff-Based Music Training Enhances Children’s Manual Dexterity and Bimanual Coordination
Source: Front Psychol. 2018 Dec 21;9:2616. doi: 10.3389/fpsyg.2018.02616 (PMC6308163; doi:10.3389/fpsyg.2018.02616)
Supplement: Supplementary file 1 [file Table_1.DOCX]

Supplementary Material

Orff-based music training enhances children’s manual dexterity and bimanual coordination

Marta Martins, Leonor Neves, Paula Rodrigues, Olga Vasconcelos, São Luís Castro*

*** Correspondence:** São Luís Castro: slcastro@fpce.up.pt

# Supplementary Tables

**Supplementary Table S1.** Music Training Program.

| **1. Music awareness** |
| --- |
| Auditory and visual recognition of Orff and orchestra music instruments |
| Recognition of different music genres and expression of personal interests related to them |
| Identification of basic music structures, and of emotions expressed in music |
| **2. Elementary music concepts** |
| Rhythm, melody and harmony |
| Rhythm figures, notes (whole, half, quarter, eighth and sixteenth), rests (half, quarter and eighth), and time signature (2/4, 3/4, 4/4, 6/8) |
| Beat, measure, bar line, double bar and repeat sign |
| Dynamics: *ff* to *pp*, *crescendo* and *diminuendo* |
| *Tempo*: lento, adagio, moderato, allegro, presto |
| *Tutti*, solo, duet |
| Major and pentatonic scales; treble clef; sharp and flat |
| **3. Rhythm and pitch** |
| Recognition and execution of rhythm figures including notes and rests, simple and compound rhythm patterns and ostinatos with steady and variable beat |
| Perception of pitch variations and association with body movement |
| Recognition and execution of melodic patterns |
| Recognition of pitch notes on staff (treble clef) |
| **4. Performance** |
| Individual and choir vocal performance, one to two vocal layers |
| Individual and group instrumental performance with Orff instruments (drums, xylophones and metallophones) or descant recorder (single to four-part harmony) |
| Vocal and instrumental improvisation/imitation through echo (call and response) |
| Body movement in response to tempo and dynamics variations |
| Following conductor directions (tempo, dynamics and extra cues) |
| Appropriate rehearsal behavior |

**Supplementary Table S2.** Sports Training Program.

| **1. Physical fitness (basketball-oriented)** |
| --- |
| Warm-up exercises (with and without materials, namely balls) |
| Running technique: control of body and motion, pace, and coordination (resistance, velocity running and sprinting) |
| Strength and flexibility activities |
| Exploring several ways of jumping (taking off from one foot or two feet, and landing on two feet) |
| Exploring several ways of throwing (different positions of arms and differently sized materials) |
| **2. Coordination skills** |
| Coordination of upper and lower limbs, separately and with each other |
| Eye-hand coordination |
| Eye-foot coordination |
| Complex movements of body parts and body actions, including weight transference |
| **3. Team sports: basketball** |
| Development of ball-handling skills and pair/group relays |
| Learning dribble, block, pass and shooting technique |
| Working rebounding in basketball |
| The game: rules and team practice |
| **4. Team work: tactical planning** |
| Pre-team games to explore: |
| - occupation of space |
| - cooperation |
| - companionship |
| Discussion and implementation of tactical plans |
